# Quantitative Measure of Receptor Agonist and Modulator Equi-Response and Equi-Occupancy Selectivity

**DOI:** 10.1038/srep25158

**Published:** 2016-04-27

**Authors:** Rumin Zhang, Michael Kavana

**Affiliations:** 1 Merck Research Laboratories, Department of In Vitro Pharmacology, Kenilworth, New Jersey, USA

## Abstract

G protein-coupled receptors (GPCRs) are an important class of drug targets. Quantitative analysis by global curve fitting of properly designed dose-dependent GPCR agonism and allosterism data permits the determination of all affinity and efficacy parameters based on a general operational model. We report here a quantitative and panoramic measure of receptor agonist and modulator equi-response and equi-occupancy selectivity calculated from these parameters. The selectivity values help to differentiate not only one agonist or modulator from another, but on-target from off-target receptor or functional pathway as well. Furthermore, in conjunction with target site free drug concentrations and endogenous agonist tones, the allosterism parameters and selectivity values may be used to predict *in vivo* efficacy and safety margins.

G protein-coupled receptors (GPCRs) represent the largest class of druggable targets^1^. A ubiquitous feature of GPCRs is their ability to adopt ensembles of functionally inactive and active, often signaling pathway-selective, conformational states which are further stabilized upon binding to agonists and/or allosteric modulators^2^. Such conformational plasticity, along with a diverse array of downstream effectors and adaptors, contribute to the observed pleotropic functional agonism and allosterism of GPCRs^3^. Receptor agonism and allosterism can be quantitatively analyzed using operational model (OM) or allosteric two-state model (ATSM) to derive agonist- and modulator-specific affinity and efficacy parameters^2^,^4^,^5^,^6^. A longstanding tradition of receptor pharmacology has been to use the effective concentration for 50% maximal response window (EC_50_) to measure agonist or modulator potency, analogous to the half maximal inhibitory concentration (IC_50_) concept for enzyme inhibitors or receptor antagonists. Ratios of EC_50_ or IC_50_ values are then calculated to provide a measure of selectivity. To measure agonist bias, the logarithmic difference between a pair of “intrinsic relative activity” (i.e., reference-normalized ratio of maximal response over half maximal response concentration)^7^,^8^ or “transduction coefficient” (reference-normalized ratio of efficacy over affinity)^9^,^10^ have been proposed, not without limitations^11^,^12^,^13^. However, a general method for measuring various aspects of selectivity (including agonist, modulator, receptor and signaling pathway selectivity) and the resulting bias based on ligand concentrations and receptor levels as well as all parameters of receptor agonism and allosterism has been lacking to date.

When two dose-dependent receptor functional response curves are compared, the conventional midpoint EC_50_ or IC_50_ (or any EC_x_ or IC_x_) values may not mean equal response, even though they are at half (or x%) of each curve’s own maximal window of response. The reason is that, for receptor agonism and allosterism, the maximal response can be less than the maximal system response if partial agonism, strong negative allosterism or weak positive allosterism is involved. Even when the maximal system response is achieved with full agonism and/or strong positive allosterism, the minimal response may not be the same if constitutive receptor activity and/or modulator’s efficacy is taken into account. In all of the above cases, the midpoint EC_50_ or IC_50_ or EC_x_ or IC_x_ value for each curve can mean a different relative level of response. To address this conundrum of comparison at unequal receptor functional response or occupancy and to develop both dose- and all parameters-dependent selectivity measure that is broader and more general than agonist bias, we apply the concept of null (equal response) method to the dose-response curves in receptor agonism and allosterism. Historically, a null method-based, Gaddum or Schild analysis^14^,^15^,^16^ of dose ratios (ratios of equiactive agonist concentrations) in the presence and absence of a modulator has been used to determine modulator affinity, verify the mode of binding (orthosteric or allosteric) or flag non-equilibrium or heterogeneous receptor binding. Similarly, Furchgott analysis^17^ of equiactive agonist concentrations before and after irreversible receptor inactivation has been used to analyze agonist affinity and efficacy.

We report here a quantitative and panoramic measure of agonist or modulator selectivity at equal fractional response or occupancy, termed as “equi-response” or “equi-occupancy” selectivity, respectively. Rather than resorting to tedious graphical interpolations of pairwise curves to arrive at a finite number of selectivity values, we calculate a continuous, panoramic equi-response and equi-occupancy selectivity space from all parameters measured in dose-dependent receptor agonism and allosterism assays. The affinity and efficacy parameters based on OM or equivalent ATSM can be obtained by a global curve fitting analysis of functional data (see a recent review^6^ and references cited therein for practical guidance). Nonequivalent ATSM such as receptor states model^18^,^19^ may also be used to obtain affinity and efficacy parameters with the caveat of ignoring ligand cooperativity that can be significant for strongly positive or negative allosteric modulators^20^. Equi-response and equi-occupancy selectivity can be used to differentiate not only one agonist or modulator from another, but on-target from off-target receptor or signaling pathway. Thus it provides a comprehensive way of comparing head to head two agonists, modulators, receptors or signaling pathways. This measure of selectivity based on a hitherto unpublished set of equations relating two sets of all relevant parameters represents a new development in quantitative receptor pharmacology. It has the advantage of capturing the nonobvious, subtle and/or substantial impact of all agonism and allosterism parameters in the context of fluctuating concentrations of agonist and modulator, potentially enabling predictive or translational pharmacokinetics-pharmacodynamics.

## Methods

A set of equations based on the general operational model with constitutive receptor activity and all affinity and efficacy parameters of receptor agonism and allosterism were derived to calculate equi-response or equi-occupancy selectivity for modulator or agonist as defined by various equations below. Key equations were then customized in GraphPad Prism (Table 1) or Microsoft Excel (Supplementary Dataset 1). Simulations were performed by varying various parameters.

Fractional functional response in a dose combination matrix between agonist and modulator can be described by equation (1) according to a general operational model with constitutive receptor activity^6^.


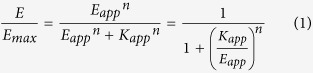


where,


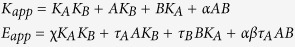


Parameters are defined exactly as previously described^6^, i.e., respectively, A and B are agonist and modulator concentrations, K_A_ and K_B_ are agonist and modulator binding affinity (dissociation constant) to the inactive receptor state, α and β are modifiers to affinity and efficacy, χ, τ_A_, τ_B_ are the efficacy parameters for receptor alone, agonist-bound receptor and modulator-bound receptor. When K_app_ = E_app_, the fractional functional response is at 50% maximal system response, allowing one to calculate the agonist and modulator dose combination matrix to achieve half maximal response.

We shall add subscript 1 and 2 to each of the parameters to denote, respectively, the first and second set of parameters for comparison. For example, K_app1_ and K_app2_ are, respectively, the first and second aggregate affinity term. Likewise, K_B1_ and K_B2_ are, respectively, the first and second modulator affinity (dissociation constant) in comparison.

To enable comparison between two cellular functional assays employing the same receptor but with different receptor levels (as in cross cell type comparison), we further introduce a receptor 1/receptor 2 ratio (RR) to normalize the second set of efficacy terms such that RR is multiplied with all efficacy terms in E_app2_ before a general, receptor level-independent comparison. This is because all efficacy parameters (χ, τ_A_, τ_B_) are directly proportional to receptor levels in the general operational model. Multiplying the second set of efficacy parameters measured at receptor level 2 with RR (receptor 1/receptor 2) would normalize these efficacy parameters as if the normalized efficacy parameters were measured with receptor 1 level instead.

While absolute response, as opposed to fractional response, may also be calculated if the maximal system response (E_max_) is known, we decide to compare fractional response to allow more general comparisons across different assays (different receptors or pathway-specific signaling readouts) with potentially different maximal system responses.

### Derivation of equi-response selectivity equations

At any identical fractional response (i.e., equi-response), the following equation holds true based on equation (1) and receptor level normalization factor (RR) described above.


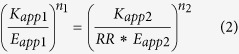


Plugging in the appropriately subscripted affinity and efficacy terms in equation (1) and solving for modulator 2 concentration (B_2_) at any fixed agonist concentration (A = A_1_ = A_2_), we obtain


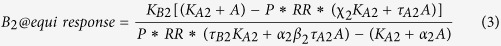


where,


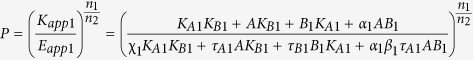


Thus, modulator equi-response selectivity, defined as B_1_/B_2_, becomes


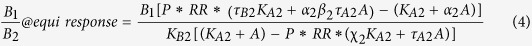


Please note when comparing two dose-response curves, only when the response from first set of data matches that from the second set, will a positive value of selectivity be calculated. A negative value suggests non-matching response levels and is arbitrarily assigned a value of zero in our selectivity plot to avoid automatic scaling that may obscure small positive values.

Alternatively, solving for agonist 2 concentration (A2) at any fixed modulator concentration (B = B1 = B2), we obtain


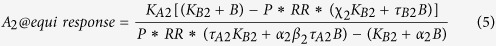


where,


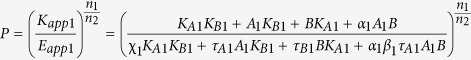


Likewise, agonist equi-response selectivity, defined as A_1_/A_2_, becomes


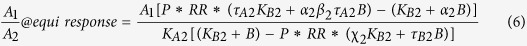


Notice both equation (4) and (6) are dependent on agonist and modulator dose combination matrix (AxB1, A1xB) as well as affinity and efficacy parameters, although equation (4) and (6) are, respectively, a modulator- or agonist-centric way of defining equi-response selectivity. When setting P = 1, the calculated equi-response selectivity from equations (3, 4, 5, 6) is for the special case of 50% maximal system response. When setting A = 0, equation (4) simplifies to the special case of modulator equi-response selectivity in the absence of an agonist, a measure of dose-dependent modulator bias. Similarly, when setting B = 0, equation (6) simplifies to the special case of agonist equi-response selectivity in the absence of a modulator, a measure of dose-dependent agonist bias by incorporating all parameters from receptor agonism.

### Derivation of equi-occupancy selectivity equations

Based on a general operational model with a slope factor of one (i.e., equivalent to the allosteric two-state model) for receptor allosterism, fractional receptor occupancy by agonist is defined as the ratio of all agonist-bound receptor species over total receptors:


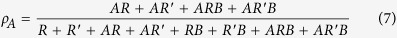


where, R, AR, BR and ARB denote free receptor, agonist-bound receptor, modulator-bound receptor, and agonist- and modulator-bound receptor, respectively. R and R′ indicate functionally inactive and active receptor, respectively.

Dividing both numerator and denominator by R and plugging in the following relationships for involved equilibria,

R′/R = χ, AR/R = A/K_A_, AR′/R = AR′/AR * AR/R = τ_A_ * A/K_A_, RB/R = B/K_B_, R′B/R = R′B/RB * RB/R = τ_B_ * R/K_B_, ARB/R = ARB/R/A/B * A * B = α * A/K_A_ * B/K_B_, AR′B/R = AR′B/ARB * ARB/R = (β * τ_A_) * α * A/K_A_ * B/K_B_

we obtain fractional receptor occupancy by agonist, modulator or both, respectively,


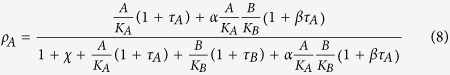



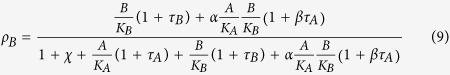



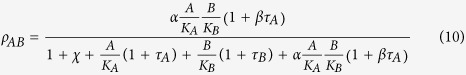


These equations allow one to calculate agonist and modulator dose combination matrix to achieve any fractional receptor occupancy by agonist, modulator or both. We will focus on the receptor occupancy by agonist in equi-occupancy selectivity calculation.

To derive equi-occupancy selectivity based on equation (8), we add subscript 1 and 2 to the first and second set of parameters and multiply receptor 1/receptor 2 ratio (RR) for set 2 efficacy parameters as similarly done above for equi-response selectivity. Thus,


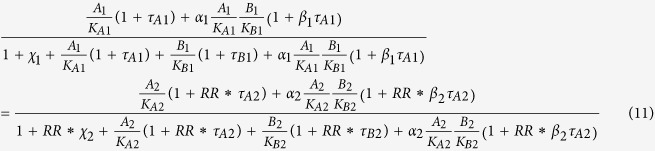


Solving for modulator 2 concentration (B_2_) at any agonist concentration (A = A_1_ = A_2_), we obtain


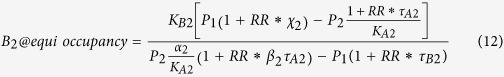


where,


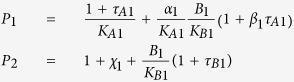


Therefore, modulator equi-occupancy selectivity, defined as B_1_/B_2_, becomes


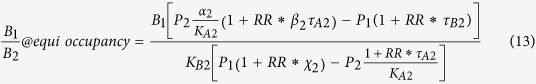


Alternatively, solving Equation (11) for agonist 2 concentration (A_2_) at any fixed modulator concentration (B = B_1_ = B_2_), we obtain


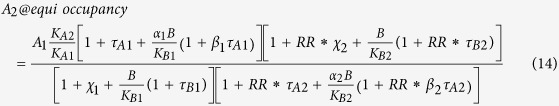


Therefore, agonist equi-occupancy selectivity, defined as A_1_/A_2_, becomes


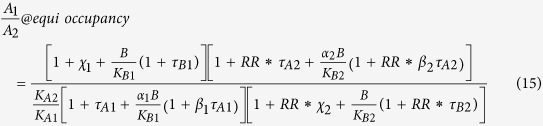


Notice that agonist concentration term drops out in equations (12), (13) and (15), suggesting modulator or agonist equi-occupancy selectivity is *independent* of specific agonist concentrations, contrary to the dependence of equi-*response* selectivity on both agonist and modulator concentrations. Thus, there is no need to calculate a special case of 50% equi-occupancy selectivity, since it is the same selectivity value at any other fractional occupancy by a given agonist. Notice also that the slope factor (n) is absent from all equi-occupancy equations because a slope factor of one for the operational model is assumed in deriving equations (7, 8, 9, 10, 11, 12, 13, 14, 15). If slope factor deviates significantly from unity, it may suggest a more complex model (such as receptor oligomerization) is needed for receptor agonism and allosterism. In that case, equi-occupancy selectivity may not as closely approximate reality as equi-response selectivity which takes into account slope factor in the calculations. When setting B = 0, equation (15) simplifies to the special case of agonist equi-occupancy selectivity in the absence of a modulator.

### Derivation of equations for deriving functional parameters from whole cell binding assay

Taking note of the definitions of EC_50_ values for various receptor species (AR, RB, ARB), we can rearrange the above equation (8) for fractional receptor occupancy by a properly labeled and detectable (e.g., radioactive or fluorescent) agonist as follows:


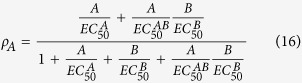


where, EC_50_ values are efficacy-corrected potency and defined as key geometric descriptors^5^:


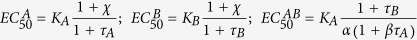


Similarly, solving equation (9), fractional receptor occupancy by a properly labeled modulator is


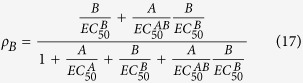


Finally, solving equation (10), fractional receptor occupancy by both agonist and modulator is


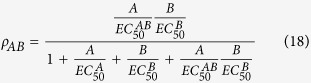


The above rearranged equations (16, 17, 18) suggest that a properly designed dose combination matrix-dependent receptor binding assay with functional whole cells may be able to allow reliable determination of EC_50_ values for all bound receptor species (AR, RB, ARB). In other words, a binding assay performed with functional whole cells can be “functional” in the sense that it uncovers efficacy-corrected potency. If such binding assay is performed on the same type of cells but with various levels of receptors then both affinity and efficacy parameters may be determined. Of course, since binding assay readout captures all the agonist- or modulator-bound receptor species (depending on which ligand is being detected), regardless of receptor functional states (inactive and active) and downstream signaling pathway selectivity, the measured efficacy parameters and EC_50_ values reflect a sub-population weighted average behavior of all functionally active species. Thus, one may not observe a strict concordance with efficacy parameters determined from a functional agonism and allosterism assay. All affinity and efficacy parameters are best determined from a properly designed functional assay.

### Error estimation for equi-response and equi-occupancy selectivity

Since the equi-response and equi-occupancy selectivity calculated according to the above derived equations 4, 6, 13, and 15 is a function of multiple parameters of receptor agonism and allosterism, the error estimate for the selectivity will be dependent on the propagation of uncertainty for all parameters. Instead of resorting to an elaborate statistical means for error estimate, we recommend the following simple and reasonable method. Since modulator or agonist selectivity is defined as a concentration ratio (B1/B2 or A1/A2) that yields equal receptor response or occupancy, the variance of selectivity is jointly determined by the variance of any such pairs of concentrations. One can fit the dose-response curves by a simple 4-parameter logistic equation to estimate the variance for pEC_50_ which usually have the largest variance. The variance of logarithm of selectivity value based on any pair of curves is then calculated as the sum of the variance. Proper antilog calculation gives rise to the estimated variance of selectivity.

## Results

### Equi-response Selectivity

Modulator or agonist equi-response selectivity is defined, respectively, as B1/B2 or A1/A2, the ratio of the first modulator or agonist concentration over the second modulator or agonist concentration when identical fractional functional response is achieved. Mathematical definitions for equi-response selectivity are found in equations (4) and (6) in section 1 of Method. Since receptor alloterism dose response curves are dependent on both agonist and modulator concentrations as well as affinity and efficacy parameters, equi-response selectivity is also dependent on the dose combination matrix and all parameters. The corresponding ready to use GraphPad Prism-based equations are listed in Table 1 and Microsoft Excel-based selectivity simulator is provided in the Supplementary Dataset 1.

**Figure 1:**
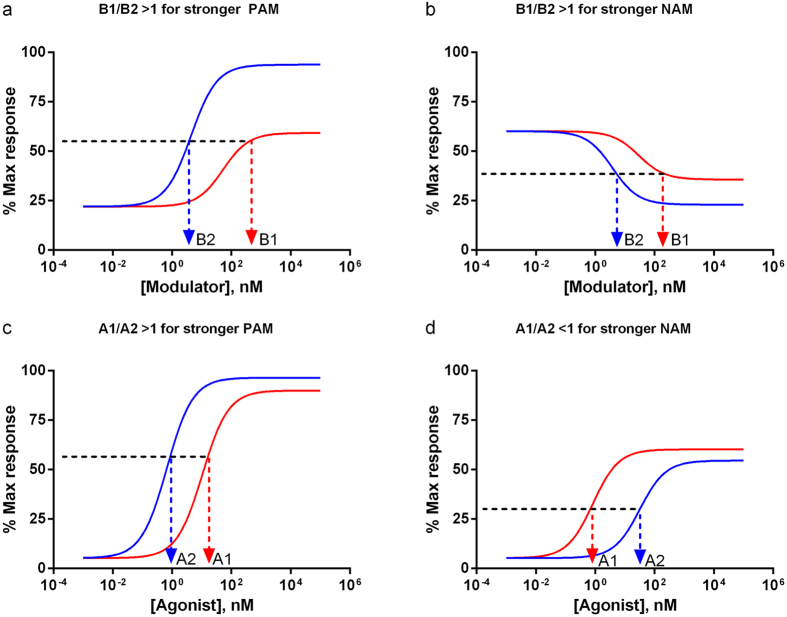
Correlation of equi-response selectivity with allosteric modulator strength. Dose response curves are compared pairwise between set 1 (red) and set 2 (blue). Equi-response selectivity value greater than one always means lower concentration indicated on the abscissa is needed for set 2 to achieve the same level of response as set 1. Notice modulator equi-response selectivity values greater than one (B1/B2 > 1) mean stronger second PAMs or NAMs (**a,b**) and agonist equi-response selectivity values greater than one (A1/A2 > 1) indicate stronger second PAM (**c**). However, it is agonist equi-response selectivity value *less than one* (A1/A2 < 1) that means a stronger second NAM, since *more* concentration of agonist 2 is needed to restore to the same response than that of agonist 1 (**d**).

Based on the above definitions, it is apparent that an equi-response selectivity value larger than one always means less concentration indicated on the abscissa is needed for the set 2 curve (blue, Fig. 1) to achieve the same response level than for the set 1 curve (red, Fig. 1). A stronger PAM or NAM as modulator 2 will report a higher than one *modulator* equi-response selectivity value (B1/B2 > 1, Fig. 1a,b). Similarly, a stronger PAM as modulator 2 will lead to a higher than one *agonist* equi-response selectivity value (A1/A2 > 1, Fig. 1c). However, a stronger NAM as modulator 2 which shifts the dose response curve to the lower right will yield a lower than one *agonist* equi-response selectivity value (A1/A2 < 1, Fig. 1d). It is important to bear in mind this negative correlation between *agonist* equi-response selectivity value and the strength of NAMs (but not PAMs). For this reason, modulator equi-response selectivity which gives a consistent meaning for both PAM and NAM may be preferred over agonist equi-response selectivity.

**Figure 2:**
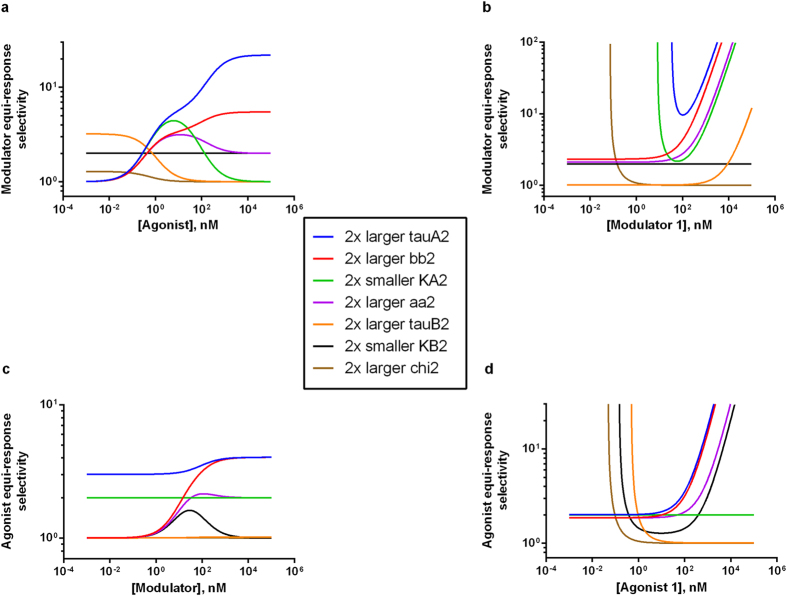
Modulator or agonist equi-response selectivity 2D plots. All simulations for 2D plots of modulator (**a,b**) or agonist (**c,d**) equi-response selectivity use the following parameters values (unless indicated otherwise in the label legend): KA1 = KA2 = KB1 = KB2 = 100 nM, chi1 = chi2 = 0.01, tauA1 = tauA2 = 3, tauB1 = tauB2 = 0.1, n1 = n2 = 1, aa1 = aa2 = 2, bb1 = bb2 = 3, RR = 1. (**a**) Modulator 1 concentration = 100 nM. (**b**) Agonist concentration = 100 nM. (**c**) Agonist 1 concentration = 100 nM, (**d**) Modulator concentration = 100 nM.

**Figure 3:**
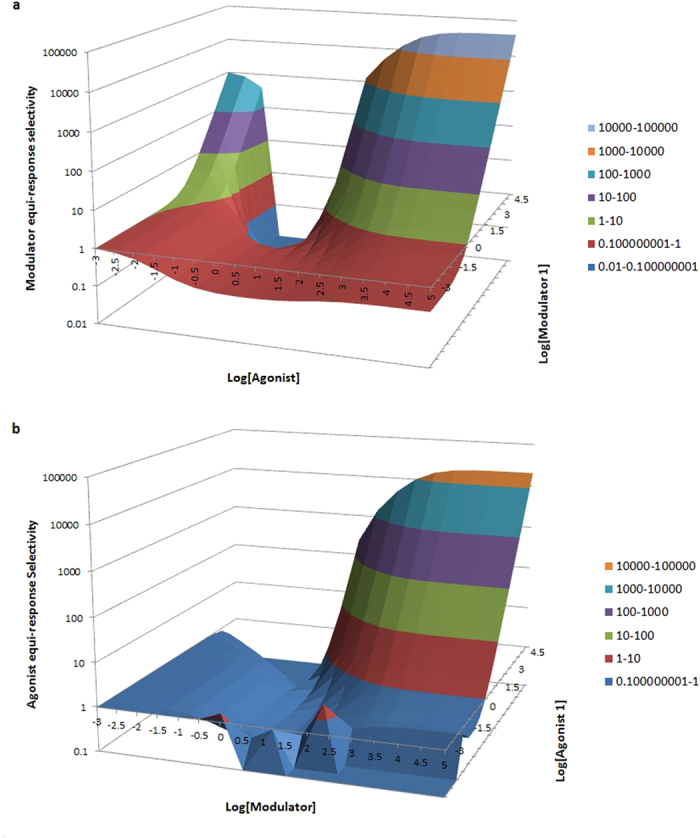
Modulator or agonist equi-response selectivity 3D plots. All simulations for 3D surface contour plots of modulator (**a**) or agonist (**b**) equi-response selectivity use the following parameters values: KA1 = KA2 = 100 nM, chi1 = chi2 = 0.01, tauA = tauA2 = 3, n1 = n2 = 1, KB1 = 50nM, KB2 = 100nM, tauB1 = 0.1, tauB2 = 0.2, aa1 = 100, aa2 = 10, bb1 = 2, bb2 = 6, RR = 1.

The first and the second agonist or modulator for comparison can be two different molecules in the same receptor and assay context or the same molecule but in different receptor or assay context. Thus, equi-response selectivity based on equations (4) and (6) and Figs 2 and 3 can be used to compare two modulators, agonists, receptors or signaling pathways (more on this multifaceted selectivity later).

In Fig. 2a, the *modulator* equi-response selectivity (i.e., B1/B2, the ratio of modulator concentration in the first curve over modulator concentration in the second curve) is calculated from equation (4) and plotted against a range of common agonist concentrations at any given first modulator concentration. In Fig. 2b, the same selectivity is calculated similarly but plotted instead against a range of the first modulator concentrations at any given common agonist concentration.

To gauge the impact of each parameter on the equi-response selectivity, we systematically varied the second set of parameters by 2-fold, as indicated by Fig. 2 legend labels. It is apparent that the second efficacy parameter (τ_A2_) has the greatest impact. Next to that is the second efficacy modifier value (β_2_) whose increase gives superior equi-response selectivity than a comparable second affinity modifier value (α_2_) increase at saturating agonist (Fig. 2a) and modulator 1 (Fig. 2b) concentrations. This superior impact of β over α on equi-response selectivity can be predicted by equation (4) (note the subtraction term containing α_2_ in the numerator) and is also consistent with the previously reported efficacy-based “beta supremacy” phenomenon for receptor allosterism^6^. The second agonist affinity parameter (K_A2_) has a bell-shaped effect on equi-response selectivity. In contrast, larger second modulator intrinsic efficacy (τ_B2_) causes equi-response selectivity going down asymptotically with increasing agonist concentrations (Fig. 2a) and up perpetually with increasing first modulator concentrations (Fig. 2b). The fold of second modulator affinity (K_B2_) change, all else being equal, quantitatively predicts modulator equi-response selectivity independent of agonist or modulator concentrations, as predicted by equation (4). The second basal level receptor activity (χ_2_) has minimal impact only at the lower agonist concentration end.

Similarly, in Fig. 2c, the *agonist* equi-response selectivity (i.e., the ratio of first agonist concentration over second agonist concentration) is calculated from equation (6) and plotted against a range of common modulator concentrations at any given first agonist concentration. In Fig. 2d, the same selectivity is similarly calculated but plotted instead against a range of the first agonist concentrations at any given common modulator concentration. Similar to Fig. 2a,b, τ_A2_ has the greatest impact. β value changes exert a greater effect on agonist equi-response selectivity than α value change of comparable magnitude. Second modulator affinity (K_B2_) gives a bell-shaped impact while the second modulator efficacy parameter (τ_B2_) has some impact at the low end of agonist dose. Second agonist affinity (K_A2_) changes quantitatively predict agonist equi-response selectivity independent of agonist or modulator concentrations, as predicted by equation (6). The second basal level receptor activity (χ_2_) has minimal impact only at the lower agonist concentration end.

In real life, more than one parameter as well as the agonist and modulator concentrations will change, giving rise to a myriad of potential equi-response selectivity plots based on additional simulations using the simulator in Supplementary Dataset 1. For example, when all four modulator-specific parameters are changed (Fig. 3), the 3D contour plot of *modulator* equi-response selectivity shows complex agonist and modulator 1 dose-dependent changes (e.g., saddle like pattern in Fig. 3a, where higher selectivity is observed in the low and high end of agonist concentrations, but lower selectivity in the mid-range). The same changes with all four modulator-specific parameters coupled to agonist 1 and modulator dose combination matrix yield a different 3D contour plot of *agonist* equi-response selectivity as shown in Fig. 3b, where higher selectivity is observed with high agonist and high modulator concentrations. Thus it is important to keep in mind if modulator or agonist equi-response selectivity is being discussed due to the different definitions by equation (4) and (6). As discussed earlier, *modulator* equi-response selectivity has the virtue of giving consistent meaning for both PAM and NAM (Fig. 1a,b), it is more preferable than *agonist* equi-response selectivity (Fig. 1c,d). For completeness, however, the Excel-based simulator in Supplementary Dataset 1 automatically generates 3D plots in two angular views for both modulator and agonist equi-response selectivity.

As pointed out earlier, equi-response selectivity can have four aspects of selectivity. First, equation (4) and Figs 2a,b and 3a can be used to compare two *modulators* in the same assay (i.e., signaling pathway readout) with the same receptor and agonist. In this case, the two sets of agonist-specific parameters should be identical as input values. This is most useful to gauge the relative merit or structure-activity relationship (SAR) ranking of two modulators during lead optimization of drug discovery. Second, equation (6) and Figs 2c,d and 3b can be used to compare two *agonists* for their relative concentrations needed to achieve identical response in the same assay with the same receptor in the absence or presence of a modulator (but bear in mind the opposite meaning of agonist equi-response selectivity for PAM and NAM strength, as indicated in Fig. 1c,d).

Third, equation (4) and Figs 2a,b and 3a may also be used to compare two *receptors* (such as in two different cell lines with different receptor types or subtypes) in the same assay (i.e., signaling pathway readout) with the same modulator and agonist. In this case, the receptor ratio (RR = receptor 1 level/receptor 2 level) should be set to the best estimate value before selectivity can be accurately calculated. The receptor ratio, if not experimentally measured yet (such as by radioligand binding assays), may be provisionally varied around unity to predict potential overall trends of selectivity. Alternatively, a reference versus test modulator equi-response selectivity in one cell line is further compared with the selectivity in another by legitimately setting RR = 1 for either cell line. The ratio of two selectivity values (i.e., ratio of two ratios), analogous in spirit to the reference-normalized agonist bias calculations discussed in Introduction, may be used to gauge receptor bias when the relative receptor level (R1/R2) across two cell lines is unknown. This is most useful to measure on-target vs. off-target receptor selectivity, a measure of *off*-target toxicity when the off-target activity causes a side effect. Similar comparison between two receptors can be made with an agonist-centric view (A1/A2, with or without a modulator present) using equation (6) and Figs 2c,d and 3b, in which case one must remember the opposite meaning of agonist equi-response selectivity for PAM and NAM strength.

Fourth, equation (4) and Figs 2a,b and 3a can be further used to compare two *assays* (two signaling pathway readouts) with the same receptor, modulator and agonist. This is most useful to estimate the on-pathway vs. off-pathway selectivity for the same receptor, a measure of *on*-target toxicity when one of the pathways produces a side effect. Likewise, equation (6) and Figs 2c,d and 3b can be used to compare two assays with the same receptor and agonist in the absence or presence of a modulator, again mindful of the opposite meaning of agonist equi-response selectivity for PAM and NAM strength.

### Equi-occupancy Selectivity

**Figure 4:**
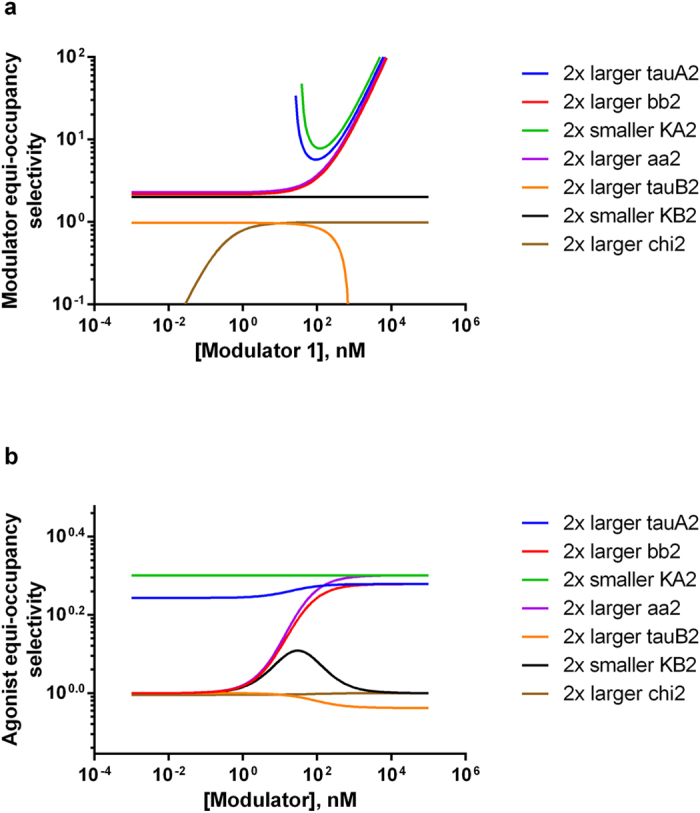
Modulator or agonist equi-occupancy selectivity 2D plots. All simulations for modulator (**a**) or agonist (**b**) equi-occupancy selectivity plots use equation (13) for (**a**) and equation (15) for (**b**) and the following parameters values (unless indicated otherwise in the label legend): KA1 = KA2 = KB1 = KB2 = 100 nM, chi1 = chi2 = 0.01, tauA1 = tauA2 = 3, tauB1 = tauB2 = 0.1, n1 = n2 = 1, aa1 = aa2 = 2, bb1 = bb2 = 3, RR = 1. Modulator concentrations are varied over 8 orders of magnitude.

For completeness and exploring potential complementarity with equi-response selectivity, we have also derived equations (13) and (15) in section 2 of Method for GraphPad Prism-based (Table 1) and Microsoft Excel-based (Supplementary Dataset 1) calculation and plotting of modulator or agonist equi-occupancy selectivity. Equi-occupancy selectivity is defined as the ratio of the first modulator or agonist concentration over the second modulator or agonist concentration (B1/B2, or A1/A2) when identical receptor occupancy by agonist (with and without modulator) is achieved. Illustrative examples of the calculations are shown in Fig. 4.

During equation derivation and data simulation, we realize that equi-occupancy selectivity is *independent* of agonist concentrations. This is understandable since fractional occupancy curves plotted as a function of agonist concentrations always span between the same two asymptotes of 0% and 100% and are shifted horizontally to the left or right for one or two different agonists in the absence or presence of a common modulator (simulations not shown). In other words, identical horizontal spacing between a given pair of such curves, thus identical agonist equi-occupancy selectivity (A1/A2), is maintained across all agonist concentrations. Less intuitively, when fractional occupancy curves are plotted instead as a function of modulator concentrations in the presence of a common agonist, the curves will have different asymptotes for different agonist concentrations. The spacing between any pair of curves at a given agonist concentration, thus modulator equi-occupancy selectivity (B1/B2), will change with modulator concentrations but will still remain the same across all agonist concentrations (simulations not shown). Given the agonist concentration independence, the agonist or modulator equi-occupancy selectivity is plotted against the concentrations of the first modulator (Fig. 4a) or a common modulator (Fig. 4b), respectively. As a consequence of agonist concentration-independence, there is no 3D surface contour plot for equi-occupancy selectivity. Similarly as discussed above, this kind of plots may be used to compare equi-occupancy selectivity for two modulators, agonists, receptors or assays.

The second agonist affinity (K_A2_) and efficacy (τ_A2_) have the greatest impact on equi-occupancy selectivity (Fig. 4a,b). In contrast to equi-response selectivity, affinity modifier (α) value change has a slightly greater effect on equi-occupancy selectivity than efficacy modifier (β) value change. This is not surprising since affinity modifier changes more directly impact agonist occupancy by changing equilibria from both RB (modulator-bound receptor) and AR (agonist-bound receptor) to ARB (both agonist- and modulator-bound receptor ternary complex). However, as understood in the mathematical equivalency between the general operational model with a slope factor of one and allosteric two-state model^5^, the efficacy modifier changes shift only the subsequent equilibrium from inactive to active receptor state for ARB ternary complex where receptor is already occupied by agonist. Thus, the second set of affinity modifier (α_2_) appears outside a parenthetical summation term containing the second set of efficacy modifier (β_2_) in equations (13) and (15), giving α_2_ slightly greater impact. Since it is ultimately efficacy jointly determined by all forms of receptor species, not receptor occupancy by agonist and/or modulator only, that truly measures receptor allosterism, it is reasonable to suggest that equi-occupancy selectivity may be less useful than equi-response selectivity.

## Discussion

In light of equi-occupancy selectivity’s less usefulness than equi-response selectivity, we take note of the understanding that receptor binding assays on whole cells depend on both affinity and efficacy parameters of agonist and/or modulator^21^. A properly designed receptor binding assay performed on whole cells may enable the determination of apparent EC_50_ and possibly affinity and efficacy parameters for various receptor species (see equations (16) and (17) and text in section 3 of Method). Since only receptor occupancy by usually agonist (equation (16)) or possibly modulator (equation (17)) is measured in receptor binding assays and no downstream signaling pathway readouts are monitored, the determined EC_50_ and possibly affinity and efficacy values for various sub-populations of receptor species represent sub-population-weighted average behaviors of functionally diverse receptor species. In other words, receptor binding assays on whole cells are “functional” only in an all pathways-inclusive and average sense. Therefore, it is preferable to calculate modulator or agonist equi-response selectivity based not on binding assay parameters but on the pathway-specific and resolved affinity and efficacy parameters determined from dose combination matrix dependent functional receptor agonism and allosterism.

When full agonist and/or strong positive allosteric modulator are involved, the full agonist’s efficacy (τ_A_) and affinity (K_A_) and modulator’s efficacy modifier (β) may not be readily determined, except when receptor levels are lowered substantially to permit determination of K_A_ and, consequently, also τ_A_ at the original receptor level^6^. Receptor level dependent oligomer formation may still complicate the analysis. In those cases, the τ_A_ and K_A_ can be tentatively co-varied with τ_A_ above a reasonable threshold for full agonist (τ_A_ > 10) and the K_A_/τ_A_ ratio, or more generally, (1 + χ) * K_A_/(1 + τ_A_) kept near the EC_50_ value of this full agonist’s functional response curve. The β value can be tentatively set to unity or a value greater than one during global curve fitting so that its impact is wholly or partially subsumed by α, the affinity modifier^6^. With a finite set of combinations of these tentative values, one can calculate a set of equi-response and equi-occupancy selectivity plots to visualize the overall trends and likely boundary limits of selectivity space.

Overall, Figs 2, 3, 4 reminds us of not only all parameters-based but also dose-dependent nature of equi-response and equi-occupancy selectivity. A single selectivity value at a fixed dose combination matrix may be misleading and is clearly less comprehensive than the panoramic selectivity space described here. The *in vivo* equi-response and equi-occupancy selectivity will dynamically change, due to pharmacokinetic exposure’s waxing and waning of exogenously administered modulator, and for equi-response selectivity, also due to the concentration fluctuations of the endogenously produced and decaying agonist. Slow binding kinetics with agonist and/or modulator can further complicate the picture. When one knows the time-dependent pharmacokinetic exposure range of modulator and *in vivo* agonist concentrations, preferably at or near target sites, it is relatively straightforward to predict *in vivo* efficacy by equation (1) as well as equi-response and equi-occupancy selectivity and on-target and off-target toxicity for candidate drug molecules according to the algorithms described here. This will help to enable predictive or translational pharmacokinetics-pharmacodynamics for therapeutic receptor modulators.

## Additional Information

**How to cite this article**: Zhang, R. and Kavana, M. Quantitative Measure of Receptor Agonist and Modulator Equi-Response and Equi-Occupancy Selectivity. *Sci. Rep.* **6**, 25158; doi: 10.1038/srep25158 (2016).

## Supplementary Material

Supplementary Dataset 1

## Figures and Tables

**Table 1:** Prism equations for calculating receptor agonist and modulator equi-response and equi-occupancy selectivity 2D plots.

Selectivity	Definitions	Prism-based equations
Modulator equi-response selectivity	Equation (4): The ratio of modulator 1 concentration for receptor 1 in assay 1 over either modulator 2 concentration for receptor 1 in assay 1, or modulator 1 concentration for receptor 2 or in assay 2, yielding the same level of functional response by said agonist and modulator	A = 10^X; for Fig. 2a (replace with B1 = 10^X; for Fig. 2b)
		Kapp1 = (KA1 + A)*KB1 + (KA1 + aa1*A)*B1
		Eapp1 = (chi1*KA1 + tauA1*A)*KB1 + (tauB1*KA1 + aa1*bb1*tauA1*A)*B1
		P = (Kapp1/Eapp1)^(n1/n2)
		Selectivity = B1*[P*RR*(tauB2*KA2 + aa2*bb2*tauA2*A)-(KA2 + aa2*A)]/{[KA2 + A-P*RR*(chi2*KA2 + tauA2*A)*KB2]}
		Y = IF(Selectivity >0,Selectivity,0)
Agonist equi-response selectivity	Equation (6): The ratio of agonist 1 concentration for receptor 1 in assay 1 over either agonist 2 concentration for receptor 1 in assay 1, or agonist 1 concentration for receptor 2 or in assay 2, yielding the same level of functional response by said agonist and modulator	B = 10^X; for Fig. 2c (replace with A1 = 10^X; for Fig. 2d)
		Kapp1 = (KA1 + A1)*KB1 + (KA1 + aa1*A1)*B
		Eapp1 = (chi1*KA1 + tauA1*A1)*KB1 + (tauB1*KA1 + aa1*bb1*tauA1*A1)*B
		P = (Kapp1/Eapp1)^(n1/n2)
		Selectivity = A1*[P*RR*(KB2*tauA2 + aa2*bb2*tauA2*B)-(KB2 + aa2*B)]/[KA2*(KB2 + B)-P*RR*KA2*(chi2*KB2 + tauB2*B)]
		Y = IF(Selectivity >0,Selectivity,0)
Modulator equi-occupancy selectivity	Equation (13): The ratio of modulator 1 concentration for receptor 1 in assay 1 over either modulator 2 concentration for receptor 1 in assay 1, or modulator 1 concentration for receptor 2 or in assay 2, yielding the same level of receptor occupancy by said agonist	B1 = 10^X; for Fig. 4a
		P1 = (1 + tauA1)/KA1 + aa1*B1*(1 + bb1*tauA1)/KA1/KB1
		P2 = 1 + chi1 + B1/KB1*(1 + tauB1)
		Selectivity = B1*[P2*aa2/KA2*(1 + bb2*tauA2*RR)-P1*(1 + tauB2*RR)]/KB2/[P1*(1 + chi2*RR)-P2*(1 + tauA2*RR)/KA2]
		Y = IF(Selectivity >0,Selectivity,0)
Agonist equi-occupancy selectivity	Equation (15): The ratio of agonist 1 concentration for receptor 1 in assay 1 over either agonist 2 concentration for receptor 1 in assay 1, or agonist 1 concentration for receptor 2 or in assay 2, yielding the same level of receptor occupancy by said agonist	B = 10^X; for Fig. 4b
		Top = (1 + chi1 + B/KB1*(1 + tauB1))*(1 + tauA2*RR + aa2*B/KB2*(1 + bb2*tauA2*RR))
		Bottom = KA2/KA1*(1 + tauA1 + aa1*B/KB1*(1 + bb1*tauA1))*(1 + chi2*RR + B/KB2*(1 + tauB2*RR))
		Selectivity = Top/Bottom
		Y = IF(Selectivity >0,Selectivity,0)
